# Transcription through enhancers suppresses their activity in *Drosophila*

**DOI:** 10.1186/1756-8935-6-31

**Published:** 2013-09-26

**Authors:** Maksim Erokhin, Anna Davydova, Alexander Parshikov, Vasily M Studitsky, Pavel Georgiev, Darya Chetverina

**Affiliations:** 1 Department of the Control of Genetic Processes, Institute of Gene Biology, Russian Academy of Sciences, 34/5 Vavilov St., Moscow 119334, Russia; 2 LIA 1066, Laboratoire Franco-Russe de recherche en oncologie, Moscow 119334, Russia; 3 Laboratory of Epigenetic Regulation of Transcription, Institute of Gene Biology, Russian Academy of Sciences, 34/5 Vavilov St., Moscow 119334, Russia; 4 Department of Pharmacology, UMDNJ-Robert Wood Johnson Medical School, Piscataway, NJ 08854, USA

**Keywords:** Chromatin enhancer, Transcription interference, Transcription regulation, Enhancer suppression, Pass-through transcription

## Abstract

**Background:**

Enhancer elements determine the level of target gene transcription in a tissue-specific manner, providing for individual patterns of gene expression in different cells. Knowledge of the mechanisms controlling enhancer action is crucial for understanding global regulation of transcription. In particular, enhancers are often localized within transcribed regions of the genome. A number of experiments suggest that transcription can have both positive and negative effects on regulatory elements. In this study, we performed direct tests for the effect of transcription on enhancer activity.

**Results:**

Using a transgenic reporter system, we investigated the relationship between the presence of pass-through transcription and the activity of *Drosophila* enhancers controlling the expression of the *white* and *yellow* genes. The results show that transcription from different promoters affects the activity of enhancers, counteracting their ability to activate the target genes. As expected, the presence of a transcriptional terminator between the inhibiting promoter and the affected enhancer strongly reduces the suppression. Moreover, transcription leads to dislodging of the Zeste protein that is responsible for the enhancer-dependent regulation of the *white* gene, suggesting a 'transcription interference’ mechanism for this regulation.

**Conclusions:**

Our findings suggest a role for pass-through transcription in negative regulation of enhancer activity.

## Background

The development of multicellular organisms involves differentiation of various cell types, which is achieved by the establishment of requisite spatial and temporal patterns of gene expression. Regulation of transcription is a highly complex process involving different regulatory DNA elements, enhancers in particular. Enhancers are positive DNA sequences containing multiple binding sites for a variety of transcription factors. These regulatory elements can activate genes over long distances, up to several tens of thousands of base pairs, and act independently of the distance and orientation with respect to the promoters of target genes [1,2].

A number of experiments performed to date indicate that a major portion of the genome is being transcribed and that a large percentage of the transcripts are accounted for by long non-protein-coding sequences (lncRNAs), either in mammals or in *Drosophila*[3-6]. Recent data suggest that many of lncRNAs have important roles in the regulation of transcription [7]. However, it was found that the expression of lncRNA clusters did not correlate absolutely, either positively or negatively, with the expression of the nearest mRNAs [8]. For instance, transcripts detected in the *Drosophila bithorax* complex correlate with the repressed state of the locus [9]. In vertebrates, many clusters of imprinted genes contain lncRNAs, and some of them have been implicated in the transcriptional silencing [10]. Similarly, the X chromosome inactivation relies on the expression of a lncRNA named Xist [11]. There is also evidence that a lncRNA expressed from the HOXC locus may negatively affect the expression of genes in the HOXD locus, which is located on a different chromosome [12].

On the other hand, there are data indicative of a positive role of lncRNAs. For example, it has been shown that intergenic transcription through the PRE element counteracts silencing [13]. Some of non-coding RNAs proved to have a positive influence on expression of neighboring protein-coding genes [14]. Moreover, there is a large class of mammalian lncRNAs originating from and/or near the enhancers, named eRNAs. They are associated with active enhancers, and the resulting bidirectional eRNAs can be spliced and polyadenylated. However, regulatory functions of eRNAs remain unknown [15-17].

The detailed mechanism of the lncRNAs action is also not clear. One possibility is that these transcripts can recruit different enzymatic complexes and act as molecular scaffolds [18]. Another possibility involves the mechanism of 'transcription interference’ in which the moving RNA pol II complex can disturb protein complexes associated with DNA [19,20]. For example, transcription across the yeast SER3 promoter interferes with the binding of activators, resulting in gene repression [21]. Another illustration from the yeasts is the dislodging of Rap1 and Gcr1 factors from the ADH1 promoter by non-coding intergenic RNA ZRR1 [22].

In order to evaluate the possible role of intergenic transcription in modulation of enhancer action, we have examined the effect of transcription on the activity of *yellow* and *white* gene enhancers using transgenic reporter systems. Here, we present evidence that intergenic transcription counteracts the ability of enhancers to stimulate the promoter of the target gene. Moreover, transcription leads to displacement of the Zeste protein that is required for activity of the enhancer that stimulates *white* expression in the eyes.

## Results

### Transcription suppresses the activity of the enhancer that stimulates *white* gene expression in the eyes

To test the role of transcription in modulation of enhancer action, we used the *yellow* and *white* genes. The *white* gene is required for eye pigmentation, with the eye-specific enhancer being responsible for the high level of its transcription [23]. The *yellow* gene is responsible for dark pigmentation of the larval and adult cuticle and its derivatives. Two upstream enhancers stimulate its expression in the body cuticle and wing blades [24,25]. At first, we examined the effect of transcription on the activity of the eye enhancer of the *white* gene.

As a test system, we chose the P-element-based transgenic integration providing the possibility to obtain, in parallel, several independent transgene insertions in different genome locations. To control the potential position effect, the main elements in all constructs used in functional tests were flanked by frt or lox sites for Flp- and Cre-recombinase, respectively. The presence of the frt and lox sites allowed us to delete the flanked DNA fragments and to compare the expression of the reporter gene before and after the deletion of the regulatory elements in one genome position.

**Figure 1:**
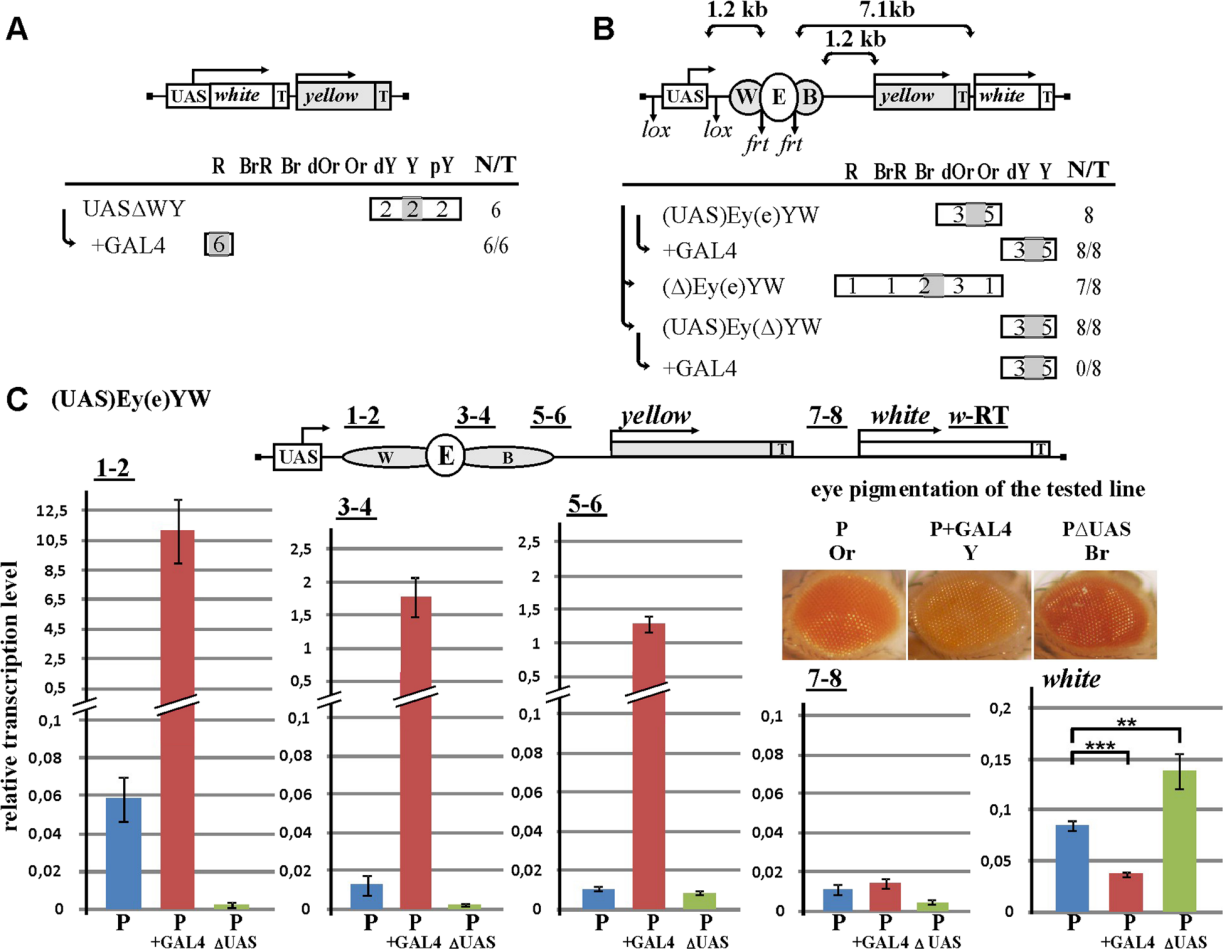
**The upstream UAS promoter suppresses activity of the *white *enhancer. (A)** UAS∆WY transgenic lines. The UAS promoter drives expression of the *white* gene; *yellow* gene was used as marker to select transgenes. “T” on the 3’-side of genes indicates terminators of transcription. Below the maps, phenotypes of parental lines and those after induction of GAL4 expression (+GAL4) are shown. The color scale for *white* is indicated above the horizontal line. Only the range of grades that were actually recorded in the flies is shown. Each entry in the frame is the number of transgenic lines with the corresponding pigmentation grade; the *shaded region* in each frame indicates the “mean level.”*T* is the total number of lines examined; for derivative constructs, *N* is the number of lines where the phenotype changed as compared with the parental construct. **(B)** (UAS)Ey(e)YW lines. The UAS promoter drives transcription through the eye enhancer (*E*) of the *white* gene, placed between wing (*W*) and body (*B*) enhancers of the *yellow* gene. *Downward arrows* indicate lox and frt sites. Below the maps are the expression data for the parental construct and for those derived after *in vivo* excision of the elements. **(C)** Quantification of (UAS)Ey(e)YW transcripts by RT-qPCR. Positions of primer pairs (1-2, 3-4, 5-6, 7-8) are indicated. Individual transcript levels were normalized relative to *ras64B* for the amount of input cDNA. The transgenic material of pupae was obtained from crosses between (P) homozygous parental line and *yw*^*1118*^ line, (P + GAL4) homozygous parental line and GAL4-expressing line, or (P∆UAS) homozygous derivative line with deleted UAS promoter and *yw*^*1118*^ line. *Error bars* indicate standard deviations. Statistical significance was analyzed using the Student’s t-test and expressed as a *P*-value. **P< 0.01; ***P< 0.005. Photographs show eye pigmentation in the heterozygous parental line and its derivatives used in RT-qPCRs.

To induce transcription, we selected the UAS promoter consisting of the minimal *hsp70* promoter (from –43 to +204 bp) and five binding sites for the GAL4 activator [26]. To confirm the ability of this promoter to drive transcription in the eyes, we constructed transgenic lines UAS∆WY in which the *white* gene was expressed under control of the UAS promoter (Figure 1A). In the absence of GAL4 stimulation, flies carrying the *white* transgene under control of the UAS promoter displayed eye color phenotypes ranging from pale yellow to dark yellow, which indicated that the UAS promoter only weakly drove *white* transcription in the eyes. Induction of this promoter by GAL4 resulted in flies with the red eye color corresponding to a high level of *white* expression.

Next, we tested whether the transcription process could influence the activity of eye enhancer ((UAS)Ey(e)YW construct) (Figure 1B). Hereafter, parentheses in construct designations enclose the elements flanked by the frt or lox sites. The *yellow* gene was used as a spacer sequence. As a result, the distance between the eye enhancer and the *white* promoter was 7.1 kb. The eye enhancer (“e”) flanked by frt sites was inserted in direct orientation relative to the *white* gene in the genomic position between the wing and body enhancers of the *yellow* gene. The UAS promoter flanked by lox sites was inserted upstream of the enhancers. In all transgenic lines tested, flies had moderate levels of eye pigmentation, suggesting partial suppression of the eye enhancer. In most of the lines, however, eye pigmentation increased significantly after deletion of the UAS promoter ((∆)Ey(e)YW). Thus, the eye enhancer was partially suppressed by the UAS promoter in the absence of GAL4. Induction of the UAS promoter by GAL4 led to complete suppression of the eye enhancer, resulting in eye phenotypes identical to those observed in the absence of enhancers ((UAS)Ey(∆)YW).

At the next step, we performed RT-qPCR analysis of RNA isolated from heterozygous mid-late pupae (the stage of high *white* expression) of the one transgenic line containing the (UAS)Ey(e)YW parental (P) construct and from its derivatives with GAL4 activator (P + GAL4) or with deletion of UAS promoter (P∆UAS). The results showed that the level of *white* transcription correlated well with the phenotypic data (Figure 1C). Transcription from the UAS promoter was relatively weak in the absence of GAL4 but increased to a high level (approximately 200-fold) upon induction by GAL4. The increased transcription was detected both upstream and downstream of the *white* enhancer but not downstream of *the yellow* terminator sequences. In agreement with phenotypic data, the level of *white* gene transcription (relative to parental lines) was reduced upon GAL4 induction but increased after deletion of the UAS promoter. A decrease in transcription level was observed downstream of *yellow* enhancers. This could be explained by the presence of AATAAA in the enhancer sequences, which could contribute to transcription termination.

**Figure 2:**
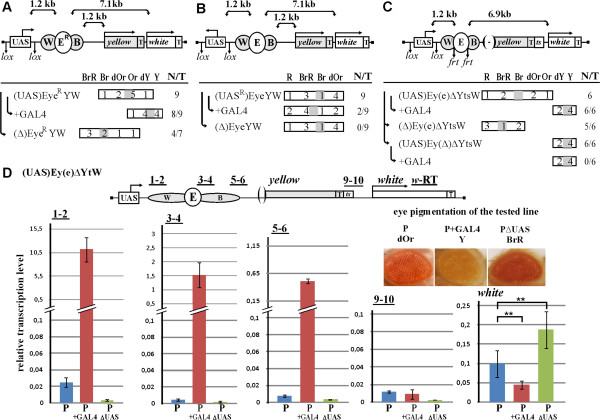
**Pass-through transcription is responsible for suppression of the eye enhancer. (A)** (UAS)Eye^R^YW transgenic lines; the eye enhancer is inserted in the opposite orientation. **(B)** (UAS^R^)EyeYW transgenic lines; the UAS promoter drives transcription in the direction from the enhancers. **(C)** (UAS)Ey(e)∆YtsW transgenic lines with deletion of the *yellow* gene promoter (indicated by the absence of an upstream arrow and by *parentheses* in front of the gene on the scheme); “*ts*” is the core 222-bp SV40 terminator. **(D)** Quantification of (UAS)Ey(e)∆YtsW transcripts by RT-qPCR. Positions of primer pairs (1-2, 3-4, 5-6, 9-10) are indicated. RT-qPCR was conducted on mRNAs isolated from transgenic lines at the mid-late pupae stage. *Error bars* indicate standard deviations. For other designations, see Figure 1.

Next, we tested the importance of eye enhancer orientation in the construct for its sensitivity to transcription from the UAS promoter ((UAS)Eye^R^YW) (Figure 2A). Eye pigmentation in transgenic flies increased after deletion of this promoter ((∆)Eye^R^YW); at the same time, induction of transcription resulted in complete inactivation of the eye enhancer. Thus, the enhancer orientation proved to be not important for the observed suppressive effect of transcription.

The suppression of the eye enhancer could be explained either by transcription through the enhancers or by competition for the enhancer between the UAS and *white* promoters. To determine the role of transcription in suppression of the eye enhancer, we inserted the UAS promoter in the opposite orientation ((UAS^R^)EyeYW) (Figure 2B) and found that all the resulting transgenic lines had an almost wild-type level of eye pigmentation, which did not decrease after either deletion of the UAS promoter ((∆)EyeYW) or induction of transcription by GAL4. These results contradict the promoter competition model, since the opposite orientation of the *white* and UAS promoters should not affect their ability to compete for the eye enhancer. Thus, transcription leads to suppression of the eye enhancer.

In the transgenic lines described above, the eye enhancer should stimulate *white* across the *yellow* promoter, which could reduce the activity of the enhancer and affect the observed result of intergenic transcription. To test this possibility we made the construct with the deleted *yellow* promoter (Figure 2C). The core 222-bp SV40 terminator (ts) fragment was added downstream of the *yellow* terminator to stabilize it. In general, lines with deletion of the *yellow* promoter showed darker eye pigmentation (cf. Figures 1B and 2C), providing indirect evidence for the ability of the *yellow* promoter to partially insulate the eye enhancer. However, deletion of the UAS promoter increased eye pigmentation in most of the transgenic lines tested, suggesting that the low level of transcription produced by the UAS promoter was still sufficient for affecting the activity of the eye enhancer (Figure 2C). As expected, induction of strong transcription by GAL4 completely repressed the eye enhancer.

One of the transgenic lines with this construct and its derivatives was selected for RT-qPCR analysis (Figure 2D). As in the previously tested line with the (UAS)Ey(e)YW construct, weak transcription from the UAS promoter increased drastically (approximately 440-fold) upon induction by GAL4. Once again, we observed a significant decrease in transcription downstream of the enhancers, suggesting partial termination of transcription in this region. In agreement with phenotypic data, the level of *white* gene transcription was reduced upon GAL4 induction but increased after deletion of the UAS promoter (Figure 2D). Thus, promoter of the *yellow* gene in this system did not affect the suppressive effect of transcription from the UAS promoter.Taken together, these results suggest that the ability of the eye enhancer to stimulate the *white* promoter is sensitive to pass-through transcription.

### The effect of transcription is not unique for the eye enhancer: *yellow* gene enhancers

In all transgenic lines described above, the eye enhancer was inserted between the wing and body enhancers of the *yellow* gene. We noticed that the UAS promoter weakly affected wing and body pigmentation only when it was located in direct orientation relative to the *yellow* enhancers (Additional file 1: Figure S1).

**Figure 3:**
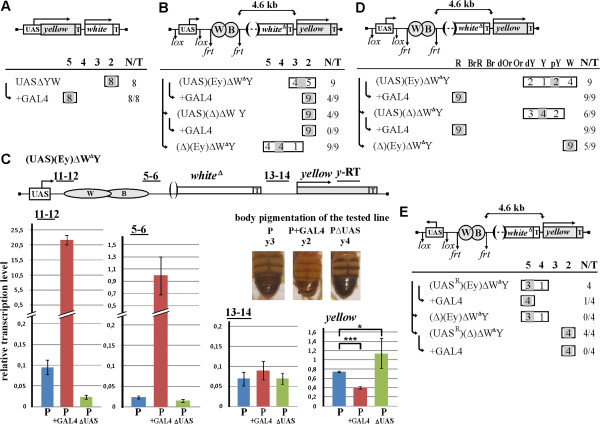
**Transcription through the *yellow *enhancers leads to their inactivation. (A)** UAS∆YW transgenic lines. The UAS promoter drives expression of the *yellow* gene. The downstream *white* gene was used as a marker to select transgenes. The color scale for *yellow* (grades 5 to 2) is indicated above the horizontal line. Grade 5 corresponds to wild-type pigmentation; grades 4 and 3 correspond to partial stimulation of the *yellow* gene by enhancers; grade 2, to the basal level of *yellow* expression in the absence of enhancers. Grade 1, corresponding to complete loss of *yellow* expression, is not shown, because no lines with such a phenotype were obtained in this study. **(B)** (UAS)(Ey)∆W^∆^Y; the UAS promoter drives transcription through the *yellow* enhancers. The *white* gene with deleted promoter was used as a spacer. **(C)** Quantification of (UAS)(Ey)∆W^∆^Y transcripts by RT-PCR. Positions of primer pairs (11-12, 5-6, 13-14) are indicated. RT-qPCR was conducted on mRNAs isolated from transgenic lines at the mid-late pupae stage. *Error bars* indicate standard deviations. *P< 0.05; ***P< 0.005. For other designations, see Figure 1. **(D)** Summarized results of eye phenotype analysis in (UAS)(Ey)∆W^∆^Y transgenic lines. **(E)** (UAS^R^)(Ey)∆W^∆^Y; the UAS promoter drives transcription in the direction from the enhancers.

To further test the suppressive effect of transcription on the *yellow* enhancers, we primarily tested the ability of the UAS promoter to drive transcription in tissues where the *yellow* gene is transcribed. For this purpose, we constructed transgenic lines carrying the *yellow* gene under control of the UAS promoter (Figure 3A). In the absence of GAL4 stimulation, the wings and bodies of transgenic flies were pigmented only slightly (grade 2), indicating a low level of *yellow* transcription, while induction of GAL4 stimulated a high level of transcription, resulting in the wild-type phenotype (grade 5) of flies.

Next, we tested whether the transcription process could influence the activity of *yellow* enhancers (Figure 3B). In the (UAS)(Ey)∆W^∆^Y construct, the *white* gene with the deleted promoter and 3’-Wari insulator (∆W^∆^) was used as a spacer inserted between the enhancers flanked by frt sites and the *yellow* promoter, so that the distance between the enhancers and the promoter was 4.6 kb. To induce transcription through the enhancers, the UAS promoter flanked by lox sites was placed immediately upstream of the enhancers.

In all transgenic lines obtained, flies had weak pigmentation of the wing and body cuticle that corresponded to the basal level of *yellow* transcription in the absence of enhancers (grade 2) or to its weak stimulation (grade 3). Induction of GAL4 resulted in the basal level of wing and body pigmentation in all transgenic lines, suggesting complete inactivation of the enhancers (Figure 3B). Deletion of the UAS promoter ((∆)(Ey)∆W^∆^Y) in the transgenic lines provided for a darker pigmentation of flies, indicating that enhancers recovered their ability to stimulate the target promoter. This result confirmed that the *yellow* enhancers were strongly suppressed in the presence of the UAS promoter. Deletion of the *yellow* enhancers ((UAS)(∆)∆W^∆^Y) resulted in the basal level of wing and body pigmentation of flies in all transgenic lines, confirming that the enhancers accounted for weak *yellow* stimulation in parental lines. These results showed that the *yellow* enhancers are very sensitive even to uninduced UAS promoter and that a high level of transcription completely inhibited their activity.

An RT-qPCR analysis of RNA isolated from mid-late pupae of one transgenic (UAS)(Ey)∆W^∆^Y line and its derivatives (the stage of high *yellow* expression) showed that transcription from the UAS promoter was relatively weak in the absence of GAL4 but increased approximately 250-fold upon induction by GAL4, with a higher transcription level being detected both upstream and downstream of the *yellow* enhancers but not downstream of *white* terminator sequences (Figure 3C). In agreement with phenotypic data, the level of *yellow* gene transcription (relative to that in the parental line) was reduced upon GAL4 induction but increased after deletion of the UAS promoter (Figure 3C). As in constructs tested previously, transcription was partially terminated on the *yellow* enhancers.

Termination of transcription by *yellow* enhancers was also observed by *white* phenotype (Figure 3D). The *white* gene contains an IRES-like element [27], which allows its expression to be used for measuring the level of upstream transcription from a distantly placed promoter. Deletion of the *yellow* enhancers from transgenic lines with the (UAS)(Ey)∆W^∆^Y construct resulted in increasing eye pigmentation, suggesting that transcription from the UAS promoter was partially terminated on these enhancers. Induction of UAS promoter by GAL4 led to red eye phenotype in transgenic flies (Figure 3D), indicating that the level of transcription downstream of the *yellow* enhancers was relatively high.

To exclude the role of promoter competition in repression of the *yellow* enhancers, we reinserted the UAS promoter in the opposite orientation ((UAS^R^)(Ey)∆W^∆^Y) (Figure 3E). The deletion of the UAS promoter or its induction by GAL4 did not lead to decrease in wing and body pigmentation, indicating that transcription through the *yellow* enhancers was responsible for their inactivation. The deletion of the *yellow* enhancers resulted in the basal level of wing and body pigmentation, confirming the role of the enhancers in *yellow* stimulation.

Taken together, the results of these experiments confirm that transcription through the *yellow* enhancers leads to their inactivation. Moreover, as in case of eye enhancer, even the very low level of transcription produced by the UAS promoter in the absence of GAL4 is sufficient for strong suppression of the enhancer activity.

### Transcription from the Ef1 promoter inhibits the activity of the enhancers

To verify that the observed effect was not unique to the UAS promoter, we tested the strong constitutive promoter of the *Elongation factor 1α48D* (Ef1).

**Figure 4:**
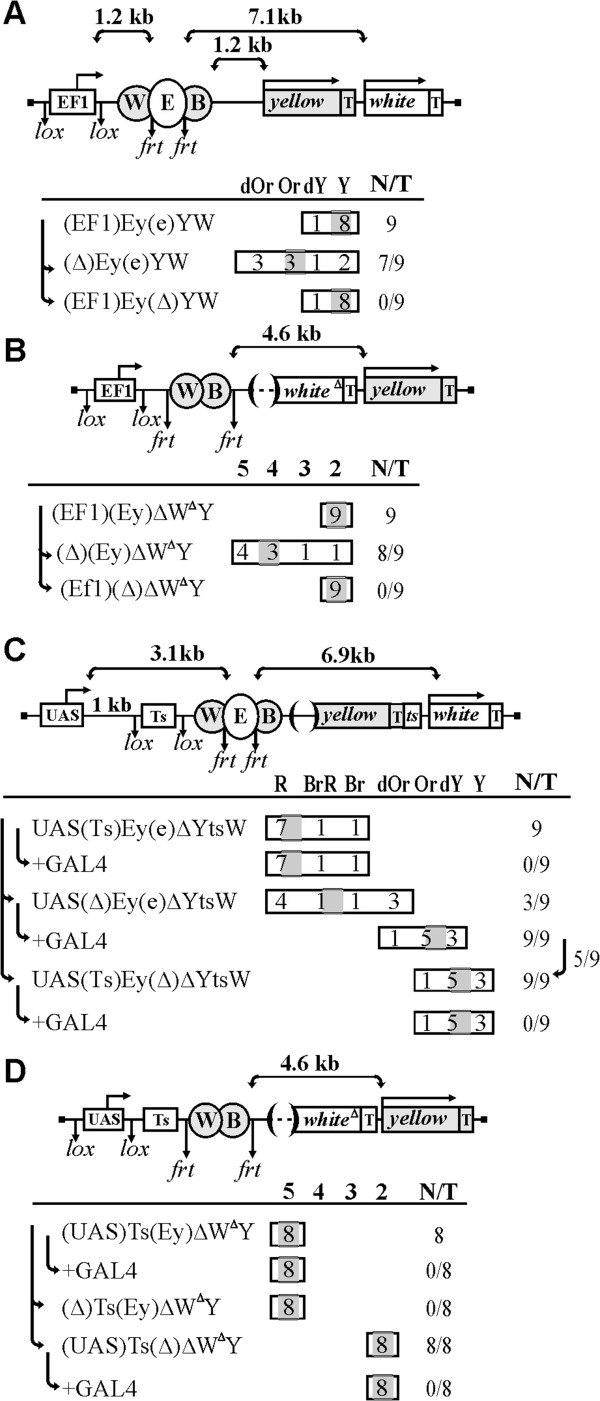
**Transcription initiated on the Ef1 promoter suppresses the enhancer activity, while the SV40 transcriptional terminator protects the enhancers from the repressive effect of transcription. (A)** (EF1)Ey(e)YW, the Ef1 promoter drives transcription through eye enhancer. **(B)** (EF1)(Ey)∆W^∆^Y, the Ef1 promoter drives transcription through *yellow* enhancers. **(C)** UAS(Ts)Ey(e)∆YtsW, the 702-bp SV40 terminator (Ts) is inserted between the UAS promoter and the eye enhancer. **(D)** (UAS)Ts(Ey)∆W^∆^Y, the 702-bp SV40 terminator is inserted between the UAS promoter and *yellow* enhancers. For other designations, see Figures 1 and 2.

The (EF1)Ey(e)YW construct was made for the eye enhancer (Figure 4A). In these transgenic lines, all flies had the weak eye pigmentation indicative of strong suppression of the enhancer. Deletion of enhancer ((EF1)Ey(∆)YW) did not change pigmentation, indicating that the enhancer is completely inactivated in the presence of Ef1 promoter, while deletion of the promoter ((∆)Ey(e)YW) resulted in darker pigmentation, restoring the ability of the enhancer to stimulate transcription. Thus, the Ef1 promoter was also found to effectively inhibit the activity of eye enhancer.

We also confirmed that the Ef1 promoter could suppress the activity of the *yellow* enhancers (Figure 4B). In (EF1)(Ey)∆W^∆^Y transgenic lines, all flies had the basal level of wing and body pigmentation, indicative of strong suppression of the *yellow* enhancers. Deletion of enhancers ((EF1)(∆)∆W^∆^Y) confirmed that they were inactive in the presence of the Ef1 promoter. At the same time deletion of the promoter ((∆)(Ey)∆W^∆^Y) restored the ability of these enhancers to stimulate transcription. Thus, the Ef1 promoter effectively inhibits the activity of *yellow* enhancers.

### The SV40 transcription terminator strongly reduces the inhibiting effect of transcription on activity of the enhancers

To further confirm that transcription is responsible for repression of enhancer activity, we used the strong transcriptional terminator from SV40 to stop transcription from the UAS promoter. To test the UAS promoter-eye enhancer pair, we inserted the 702-bp SV40 terminator flanked by lox sites between the UAS promoter and the eye enhancers (UAS(Ts)Ey(e)∆YtsW) (Figure 4C). The UAS promoter was placed at 1 kb from the SV40 terminator. As a result, the distance between the UAS promoter and the eye enhancer was 3.1 kb. As expected, induction of the UAS promoter by GAL4 did not affect the activity of the eye enhancer, confirming that SV40 terminator protects enhancer from the negative effect of the transcription. At the same time, deletion of the terminator (UAS(∆)Ey(e)∆YtsW) resulted in reduction of eye pigmentation in only three out of nine transgenic lines, suggesting that suppressive effect of transcription produced by the UAS promoter was weaker then at 1.2 kb (cf. Figures 4C and 2C). However, induction of transcription by GAL4 considerably reduced *white* expression, indicating that the eye enhancer was still sensitive to high level of transcription.

In the next construct, the SV40 terminator was inserted between the UAS promoter and the *yellow* enhancers ((UAS)Ts(Ey)∆W^∆^Y) (Figure 4D). The UAS promoter was flanked by lox sites and inserted immediately upstream of the SV40 terminator. All flies in the resulting transgenic lines had a wild-type level of wing and body pigmentation. This level decreased upon deletion of the enhancers ((UAS)Ts(∆)∆W^∆^Y), which confirmed that they were active in the parental transgenic lines. On the other hand, no changes in pigmentation were observed upon deletion of the UAS promoter ((∆)Ts(Ey)∆W^∆^Y) or its induction by GAL4, indicating that terminator effectively protected from suppressive effect of transcription. Thus, the SV40 transcription terminator protects the enhancers from repression mediated by transcription through the enhancer in transgenic lines.

### Transcription through eye enhancer leads to dislodging of Zeste protein from the enhancer

Several mechanisms may be involved in suppression of the enhancer activity by pass-through transcription. In particular, transcription may disturb binding of proteins forming active complexes on the enhancers. To test such a possibility, we compared binding of the Zeste protein to the eye enhancer in absence or presence of pass-through transcription. The *white* gene enhancer contains five binding sites for Zeste, the protein that is known to be important for communication of the eye enhancer with the *white* promoter [23,28].

**Figure 5:**
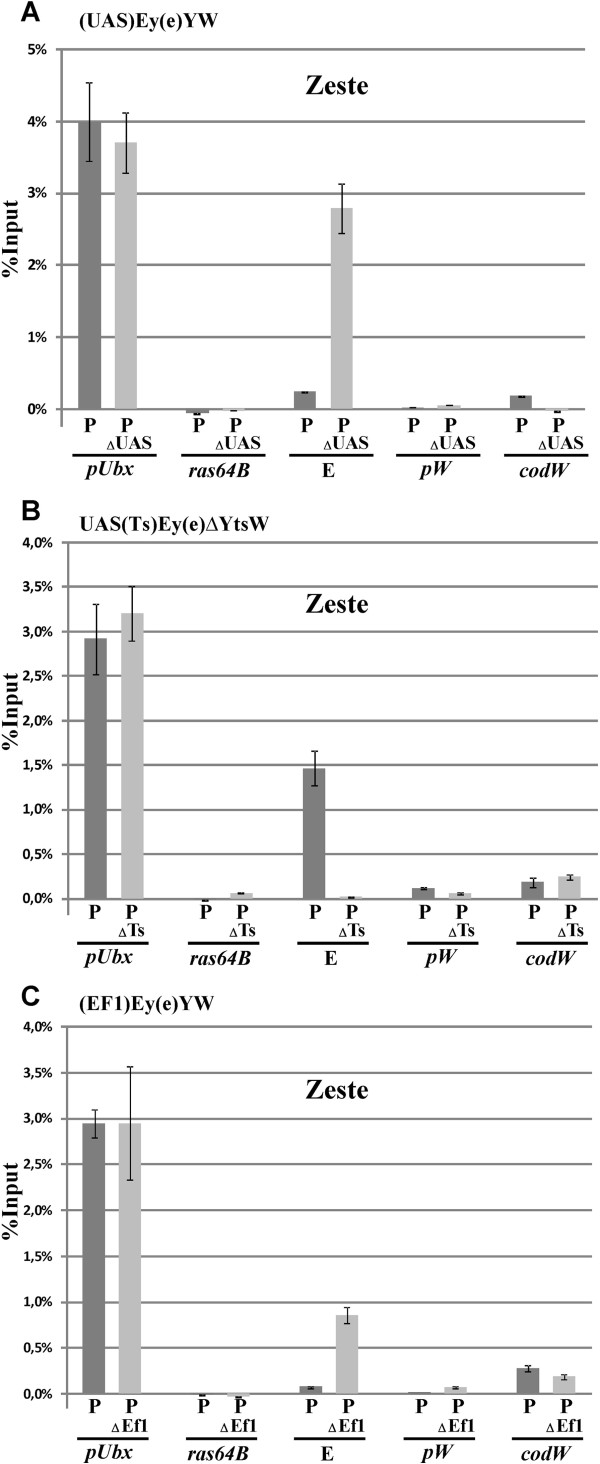
**Transcription through eye enhancer leads to dislodging of Zeste from the enhancer.** Results of ChIP with antibodies to the Zeste protein from **(A)** (UAS)Ey(e)YW, **(B)** UAS(Ts)Ey(e)∆YtsW and **(C)** (EF1)Ey(e)YW transgenic lines. Diagrams summarize the results of ChIP with specific antibodies followed by real-time PCR. The ordinate shows the percentage of target sequences in the immunoprecipitated material relative to the input (10% of total crosslinked chromatin), with the genome regions for which DNA enrichment was tested being indicated on the abscissa: *pUbx*, promoter of the *Ubx* gene, positive control; *ras64B*, negative control; E, eye enhancer of the *white* gene; *pW*, promoter of the *white* gene; *codW*, coding part of the *white* gene. “P” indicates that ChIP experiments were performed with a parental transgenic line indicated above diagram; “P∆UAS”deletion of the UAS promoter; “P∆Ts”deletion of the 702-bp SV40 terminator; “P∆EF1”deletion of the EF1 promoter. Vertical lines indicate standard deviations. All ChIP experiments were performed with chromatin isolated from heads of 2-to 5-day-old males from transgenic lines homozygous for the test construct. Background immunoprecipitation (the average normalized level after chromatin treatment with a nonspecific antibody) was subtracted from normalized specific ChIP signals (obtained with specific antibodies) at each position.

We compared binding of Zeste to the eye enhancer by chromatin immunoprecipitation (X-ChIP) assay in transgenic lines homozygous for either the (UAS)Ey(e)YW construct or its (∆)Ey(e)YW derivative obtained by deletion of the UAS promoter (Figure 5A). In all experiments, the *Ubx* promoter region known to be bound by Zeste [29] and the *ras*64B coding region were used as the positive and negative control sequences, respectively. As a result, we detected an enrichment of Zeste on the eye enhancer only in derivative transgenic line carrying the transgene lacking the UAS promoter (∆UAS) (Figure 5A), which indicated that even low level of transcription interfered with Zeste binding to the eye enhancer.

To further confirm these results, we performed the same experiment with a pair of transgenic lines carrying either the transgene with the SV40 transcriptional terminator inserted between the UAS promoter and the eye enhancer or its derivative in which this transcriptional terminator was deleted (Figure 5B). In accordance with the previous observation, the Zeste protein was detected by X-ChIP on the eye enhancer only in the presence of the transcriptional terminator.

When we tested Zeste binding to the eye enhancer in the presence or absence of the Ef1 promoter, a positive result was also obtained in the transgenic line lacking the Ef1 promoter (Figure 5C). Taken together these results suggest that transcription through the enhancer leads to dislodging of Zeste from DNA.

Finally, we tested whether pass-through transcription could recruit repression complexes to the enhancer. As shown previously, the Zeste protein is involved in regulation of transcription by Trx/PcG proteins [30,31]. Therefore, we used X-ChIP to examine binding of PcG proteins, PH, the core subunit of PRC1 [32], and Sfmbt, the core subunit of the PhoRC complex [33], to the eye enhancer in transgenic lines used for the analyzes of the Zeste binding (Additional file 2: Figure S2). As a result, we observed no enrichment with these proteins on the eye enhancer in either of these lines (Additional file 2: Figure S2). Thus, our current results do not support the model that pass-through transcription leads to recruitment of the PcG complex to the eye enhancer.

## Discussion

In this study we have demonstrated that transcription suppresses the activity of enhancers. Several mechanisms may be involved in suppression of the enhancer activity by pass-through transcription. The first possibility is 'transcription interference’ by transcription complex that can disturb the association of enhancer-bound proteins with DNA [19,20]. There are several examples demonstrating that transcription leads to dissociation of transcription factors from the promoters in yeast [21,22]. In *Drosophila*, transcription initiated from the distal promoter of the *Adh* gene can repress activity of the proximal promoter at the late developmental stages [34]. Similarly *bxd* untranslated RNAs are involved in repression of *Ubx* expression [9]. In mammalian cells, it has been shown that pass-through transcription induces dissociation of a CTCF protein from an insulator [35]. Here we have found that transcription through the *white* enhancer prevents binding of Zeste. Since this protein is critical for communication between the *white* enhancer and promoter [28], reduction of Zeste binding may account for inactivation of the eye enhancer by pass-through transcription. Such an explanation may also hold for inactivation of the *yellow* enhancers by transcription.

Suppression of enhancers might be also explained by the ability of some transcripts to recruit chromatin remodeling complexes, known in vertebrates [36]. In particular, experiments with mammals provided evidence for the recruitment of PcG complexes via ncRNA [37,38]. Our results suggest that inactivation of the *white* enhancer by transcription is not accompanied by the recruitment of the PRC1 and PhoRC complexes. However, we cannot exclude the recruitment of other chromatin remodeling complexes capable of suppressing the activity of enhancers.

Suppression of enhancer activity by transcription may play a general role in the regulation of enhancer activity. It is well known that many functionally active enhancers are located in the introns and exons of transcribed genes [39,40], and the activity of these enhancers is likely to depend on the level of interfering transcription. These types of enhancers could be regulated by a negative feedback mechanism: an increase in transcription leads to a decrease in enhancer activity, thereby preventing excessive activation of the target gene.

It is known today that some enhancer regions are transcribed into non-coding RNAs [15,16]. Similarly to feedback regulation, transcription from one cell-type-specific active enhancer can suppress the activity of neighboring enhancers that would be negatively regulated in a given group of cells or a tissue.

## Conclusions

We have analyzed the relationship between the presence of pass-through transcription and the activity of *Drosophila* enhancers using a transgenic reporter system. The results confirm that pass-through transcription suppresses the ability of enhancers to stimulate the target gene promoters. The effect of enhancer suppression has been observed in experiments with the enhancers of two different genes transcribed from two different promoters. Thus, the effect of transcription appears to be common to different *Drosophila* enhancers and not specific to the promoter driver. Even the low level of transcription induced by the UAS promoter in the absence of GAL4 activator is sufficient for noticeable inactivation of the enhancers. Accordingly, the presence of the uninduced UAS promoter leads to dislodgement of Zeste protein from the enhancer, which is important for enhancer-promoter communication.

## Methods

### *Drosophila* strains, germline transformation, and genetic crosses

All flies were maintained at 25°C on the standard yeast medium. The construct, together with a P element containing defective inverted repeats (P25.7wc) that was used as a transposase source [41], was injected into *yacw*^*1118*^ preblastoderm embryos as described [42,43]. The resulting flies were crossed with *yacw*^*1118*^ flies, and the transgenic progeny were identified by their eye or cuticle pigmentation. The transformed lines were tested for transposon integrity and copy number by Southern blot hybridization. Only single-copy transformants were included in the study.

The lines with DNA fragment excisions were obtained by crossing the transposon-bearing flies with the Flp (*w*^*1118*^*; S2CyO, hsFLP, ISA/Sco; +*) or Cre (*y*^*1*^*w*^*1*^*; Cyo, P[w+,cre]/Sco; +*) recombinase-expressing lines [44,45]. All excisions were confirmed by PCR analysis. To induce GAL4 expression, we used the modified *yw*^*1118*^; *P[w*^*¯*^*, tubGAL4]117/TM3,Sb* line (Bloomington Stock Center #5138), in which the marker *mini-white* gene was deleted as described [46].

To estimate the levels of *yellow* and *white* expression, we visually determined the degree of pigmentation in the abdominal cuticle and wing blades (*yellow*) and in the eyes (*white*) of 3-to 5-day-old males developing at 25°C, with reference to standard color scales. Pigmentation of all flies was analyzed in heterozygote. For *white*, the pigmentation scale ranges from red (*R*) in wild type, through brownish red (*BrR*), brown (*Br*), dark orange (*dOr*), orange (*Or*), dark yellow (*dY*), yellow (*Y*) and pale yellow (*pY*), to white (*W*) in the absence of expression. For *yellow*, grade 5 corresponds to wild-type pigmentation; grades 4 and 3 correspond to partial stimulation of the *yellow* gene by enhancers; grade 2, to the basal level of *yellow* expression in the absence of enhancers; grade 1, to complete loss of *yellow* expression.The pigmentation scores were independently determined by two investigators.

The details of crosses used for genetic analysis and for excision of functional elements are available upon request.

### Plasmid construction

The constructs were made on the basis of the CaSpeR vector [47]. The 5-kb *Bam*HI-*Bgl*II fragment containing the *yellow* coding region (yc) was inserted in direct orientation into the C∆ plasmid [48] cleaved with *Bam*HI (C∆-yc). The 3-kb *Sal*I - *Bam*HI fragment containing the *yellow* gene regulatory region (yr) was cloned into the pGEM7 cleaved with *Xho*I and *Bam*HI (yr-pGEM7). The *Xba*I-*Bam*HI fragment containing the *yellow* regulatory region (yr) was then cloned from the yr-pGEM7 vector into C∆-yc cleaved with *Xba*I and *Bam*HI (C∆-y). The 5-kb *Bam*HI-*Bgl*II fragment of the *yellow* gene coding region (yc) was cloned into the pCaSpeR2 plasmid (yc-C2). The production of the pCaSpeR∆700 plasmid, containing deletion of the Wari insulator at the 3’-side of the *mini-white* gene was described previously [49]. The DNA sequences of the *white* gene corresponding to the promoter region (-328 to +169) were deleted from the pCaSpeR∆700 vector [50] [∆prw-pCaSpeR∆700]. The *Aor*I-*Sma*I fragment of the *yellow* coding region (yc) with 893-bp upstream sequence lacking enhancers was then cloned from the C∆-y vector into ∆prw-pCaSpeR∆700 cleaved with *Eco*RI [∆prw-C2∆700-y(-893)]. The *Hind*III-*Eco*RI fragment containing the minimal hsp70 promoter with five GAL4 binding sites upstream of it was excised from the pUAST vector (26) and cloned into the pBluescript SK + vector between lox sites to produce the lox(UAS) plasmid.

#### UAS∆WY

The *Xba*I-*Xba*I fragment of the lox(UAS) plasmid was inserted into ∆prw-C2∆700-y(-893) cleaved with *Xba*I.

#### (UAS)Ey(e)YW

The eye enhancer (Ee) corresponding to the *white* gene regulatory sequences from position–1180 to -1849 bp relative to the transcription start site (23) was cloned into pBluescript SK + between frt sites to produce the frt(Ee) plasmid. The *Hinc*II-*Bam*HI fragment (containing the eye enhancer) of the frt(Ee) plasmid was inserted in direct orientation into the yr-pGEM7 plasmid cleaved with *Bgl*II [yr-frt(Ee)]. The *Xba*I-*Bam*HI fragment of the yr-frt(Ee) plasmid was cloned into the yc-C2 plasmid cleaved with *Xba*I and *Bam*HI [yr-frt(Ee)-yc-C2]. The *Xba*I-*Xba*I fragment of the lox(UAS) plasmid was inserted into the yr-frt(Ee)-yc-C2 plasmid cleaved with *Xba*I.

#### (UAS)Eye^R^YW

The eye enhancer without flanking frt sites was cut out of the Ee-pBluescript SK + plasmid and cloned in reverse orientation into the yr plasmid cleaved with *Bgl*II (yr-Ee^R^). The *Xba*I-*Bam*HI fragment from the yr-Ee^R^ plasmid was cloned into the yc-C2 plasmid cleaved with *Xba*I and *Bam*HI (yr-Ee^R^-yc-C2). The *Xba*I-*Xba*I fragment of the lox(UAS) plasmid was inserted into the yr-Ee^R^-yc-C2 plasmid cleaved with *Xba*I.

#### (UAS^R^)EyeYW

The eye enhancer without flanking frt sites was cut out of the Ee- pBluescript SK + plasmid and cloned in direct orientation into the yr plasmid cleaved with *Bgl*II (yr-Ee). The *Xba*I-*Bam*HI fragment from the yr-Ee plasmid containing enhancers was cloned into the yc-C2 plasmid cleaved with *Xba*I and *Bam*HI (yr-Ee-yc-C2). The *Xba*I-*Xba*I fragment of the lox(UAS) plasmid was inserted into the yr-Ee-yc-C2 plasmid cleaved with *Xba*I.

#### (UAS)Ey(e)∆YtsW

The 222-bp SV40 terminator from the pGL3basic vector (Promega) was inserted into the pBluescript SK + plasmid cleaved with *Eco*RV [SV40(s)-pSK]. The *Xho*I-*Bam*HI fragment of the SV40(s)-pSK was cloned into yc-C2 cleaved with *Bgl*II [yc-SV40(s)-C2]. The *Spe*I-*Kpn*I fragment of the (UAS)Ey(e)YW construct (containing the minimal hsp70 promoter with GAL4-binding sites and the enhancers of *yellow* and *white* genes) was inserted into yc-SV40(s)-C2 cleaved with *Bam*HI.

#### UAS∆YW

The *yellow* translation start containing *Afl*II-*Afl*II fragment was cut out of the C∆-y plasmid and inserted into the pBluescript SK + plasmid cleaved with *Eco*RV [y(ATG)-pSK]. The *Xba*I-*Xba*I fragment of the lox(UAS) plasmid was inserted into the y(ATG)-pSK plasmid cleaved with *Sma*I. The *Bam*HI*-Bam*HI fragment corresponding to the lox(UAS)-y(ATG) was cloned into the yc-C2 cleaved with *Bam*HI.

#### (UAS)(Ey)∆W^∆^Y

The *Xba*I-*Aor*I fragment containing the *yellow* gene enhancers was cut out of the yr plasmid and inserted between frt sites in pGEM-7zf [frt(yr)]. The lox(UAS) sequence was inserted into the frt(yr) plasmid cleaved with *Xba*I [lox(UAS)-frt(yr)]. The *Kpn*I–*Not*I fragment of the lox(UAS)-frt(yr) plasmid was cloned into ∆prw-C2∆700-y(-893) cleaved with *Xba*I.

#### (UAS^R^)(Ey)∆W^∆^Y

The *Sal*I-*Bam*HI fragment of the frt(yr) plasmid was inserted into the lox(UAS) plasmid cleaved with *Bam*HI [lox(U)^R^-frt(yr)]. The sequence corresponding to the lox(UAS)^R^-frt(yr) was cloned into ∆prw-C2∆700-y(-893) cleaved with *Xba*I.

#### (EF1)Ey(e)YW

The promoter of *Elongation factor 1α48D* gene was PCR-amplified with primers 5’-attgttaactgatttcgcaagc-3’ and 5’-tggatgattacactatggctgtt-3’. The PCR product was inserted into pBluescript SK + between lox sites [lox(prEf1)]*.* The resulting lox(prEf1) plasmid was sequenced to confirm that no unwanted changes had been introduced into the promoter sequence. The *Xba*I-*Xba*I fragment of the lox(prEF1) plasmid was inserted into the yr-frt(Ee)-yc-C2 plasmid cleaved with *Xba*I.

#### (EF1)(Ey)∆W^∆^Y

The *Sal*I–*Sac*II fragment of the frt(yr) plasmid was inserted into ∆prw-pCaSpeR∆700-y(-893) cleaved with *Xba*I [frt(yr)-∆prw-C2∆700-y(-893)]. The *Xba*I-*Xba*I fragment of the lox(prEf1) plasmid was inserted into frt(yr)-∆prw-C2∆700-y(-893) cleaved with *Xba*I.

#### UAS(Ts)Ey(e)∆YtsW

The *Hind*III-*Eco*RI fragment of the pUAST vector (containing the minimal hsp70 promoter with five GAL4 binding sites upstream of it) was cloned into pBluescript SK + [UAS-pSK]. The 717-bp fragment consisting of the GFP coding region (used as a spacer) was cloned into the UAS-pSK plasmid cleaved with *Hin*cII [UAS-gfp]. The *Xba*I-*Bam*HI fragment of the pUAST vector containing the 702-bp SV40 terminator was inserted into pBluescript SK + between lox sites [lox(SV40b)-pSK]. The *Xba*I–*Xba*I fragment of lox(SV40b)-pSK was cloned into the UAS-gfp plasmid cleaved with *Xho*I [UAS-gfp-lox(SV40b)]. The *Xba*I–*Kpn*I fragment containing the *yellow* and *white* enhancers was cut out of the yr-frt(Ee) plasmid and cloned into UAS-gfp-lox(SV40b) cleaved by *Bam*HI [UAS-gfp-lox(SV40b)-yr-frt(Ee)]. The *Spe*I–*Spe*I fragment of UAS-gfp-lox(SV40b)-yr-frt(Ee) was inserted into yc-SV40(s)-C2 cleaved with *Bam*HI.

#### (UAS)Ts(Ey)∆W^∆^Y

The *Xba*I–*Bam*HI fragment of the pUAST vector containing the 702-bp SV40 terminator was inserted into the lox(UAS) plasmid cleaved with *Apa*I [lox(UAS)-SV40b]. The *Xba*I-*Xba*I fragment of the lox(UAS)-SV40b plasmid was inserted into ∆prw-C2∆700-y(-893) cleaved with *Xba*I.

### RT-PCR

RNA was isolated from 20 mid-late pupae with TRI reagent (Ambion) according to the manufacturer’s instructions. Purified RNA pools were digested by RNase-free DNase I (BioLabs) and re-purified using the RNeasy Mini kit (Quagen). For reverse transcription, 3 μg of the generated RNA was incubated with ArrayScript Reverse Transcriptase (Ambion) in the presence of dNTPs, Oligo(dT) (Fermentas) and RNase inhibitor (Ambion) in the supplied reaction buffer at 42°C for 1.5 h, according to the manufacturer’s instructions. The reverse transcriptase was inactivated by heating at 95°C for 5 min. To control DNA digestion by DNase I, additional negative control experiments were performed without reverse transcriptase in the reaction mixture. The generated cDNA pools were used as templates in real-time qPCR using a C1000™ Thermal Cycler with the CFX96 real-time PCR detection module (Bio-Rad). Each PCR was performed in triplicate; cDNA pools were obtained in technical duplicate. Relative levels of mRNA expression were calculated in the linear amplification range by calibration to a DNA fragment standard curve (for genomic DNA) to account for differences in primer efficiency. The results of RT-PCR detection of *ras64B* were used to standardize the overall amount of cDNA used in PCR assays. Primers used for Q-PCR are given in Additional file 3: Table S1.

### X-ChIP

For each experiment, 200 heads from 2-to 5-day-old flies were collected. The material was homogenized in 5 ml of buffer A1 (15 mM HEPES, pH 7.6; 60 mM KCl, 15 mM NaCl, 4 mM MgCl_2_, 0.5% Triton X-100, 0.5 mM DTT) supplemented with the EDTA-free protease inhibitor cocktail (Roche, Switzerland) and formaldehyde as a crosslinking agent (final concentration 1.8%). The reaction was stopped by adding glycine (final concentration 225 mM). The homogenate was cleared by passing through 100-μm nylon cell strainer (BD Falcon) and pelleted by centrifugation at 4,000 *g,* 4°C for 5 min. After washing in three 3-ml portions of buffer A1 at 4°C (5 min each) and 3 ml of lysis buffer without SDS, the pellet was treated with 0.5 ml of complete lysis buffer (15 mM HEPES, pH 7.6; 140 mM NaCl, 1 mM EDTA, 0.5 mM EGTA, 1%Triton X-100, 0.5 mM DTT, 0.1% sodium deoxycholate, 0.1% SDS, 0.5% N-lauroylsarcosine, EDTA-free protease inhibitor cocktail) and sonicated to break chromatin into fragments with an average length of 700 bp. The material was pelleted by centrifugation at 18,000 *g* for 5 min, and the supernatant fluid was transferred to a new tube. The pellet was treated with the second 0.5-ml portion of lysis buffer, and the preparation was centrifuged at 18,000 *g* for 5 min. The two portions of the supernatant fluid were pooled, cleared by centrifuging twice at 18,000 *g* for 10 min, and the resultant chromatin extract (1 ml) was used in four ChIP experiments after preincubation with A-Sepharose or G-Sepharose (see below). One aliquot (1/10 volume) of chromatin extract after preincubation with Sepharose was kept as a control sample (Input).

ChIP experiments involved incubation with rat antibody to Zeste, rabbit antibody to Sfmbt and rabbit antibody to PH. Corresponding nonimmune IgGs were used as nonspecific antibody controls. Antibody-chromatin complexes were collected with either protein A-Sepharose (Sfmbt and PH) or G-Sepharose (Zeste) beads (Thermo Scientific). The enrichment of specific DNA fragments was analyzed by real-time qPCR, using a C1000™ Thermal Cycler with CFX96 real-time PCR detection module (Bio-Rad).Primers used in ChIP/real-time PCR analyses are listed in Additional file 4: Table S2.

### Antibodies

Antibodies against Zeste (C-end 105 aa of Zeste protein) were raised in rats. Antibodies against Sfmbt (1-348 aa of Sfmbt protein isoform B) and PH (87-521 aa of Ph-p protein isoform A) were raised in rabbits. In all cases, epitopes for antibody production were expressed as 6 × His-tagged fusion proteins in *Escherichia coli,* affinity purified on Ni Sepharose 6 Fast Flow (GE Healthcare) according to the manufacturer’s protocol and injected into rats/rabbits following the standard immunization procedure. Antibodies were affinity-purified on the same epitope as was used for immunization and tested by Western blotting from wild-type and null material or by IP to confirm their specificity (Additional file 5: Figure S3 and Additional file 6: Supplementary methods).

## Abbreviations

ChIP: Chromatin immunoprecipitation; DTT: Dithiothreitol; PcG: Polycomb group; PCR: Polymerase chain reaction; qPCR: Quantitative polymerase chain reaction; EDTA: Ethylenediaminetetraacetic acid; EGTA: Ethylene glycol-bis(2-aminoethylether)-N,N,N’,N’-tetraacetic acid; SDS: Sodium dodecyl sulfate.

## Competing interests

The authors declare that they have no competing interests.

## Authors’ contributions

ME, VS, PG and DC conceived and designed the experiments. ME, AD and DC performed cloning, fly crosses and analysis, and antigen expression. ME performed affinity purification of antibodies and ChIP analysis. ME and DC performed RT-PCR experiments. AP performed embryo injections. ME, VS, PG and DC analyzed the data and wrote the manuscript. All authors read and approved the final manuscript.

## Supplementary Material

Additional file 1: Figure S1 Summarized results of wing and body phenotype analysis in (a) (UAS)Ey(e)YW and (b) (UAS^R^)EyeYW transgenic lines. Click here for file

Additional file 2: Figure S2 Results of ChIP with antibodies to (a) Ph and (b) Sfmbt. Diagrams summarize the results of ChIP with specific antibodies followed by real-time PCR. The ordinate shows the percentage of target sequences in the immunoprecipitated material relative to the input (10% of total cross-linked chromatin), with the genome regions for which DNA enrichment was tested being indicated on the abscissa: *bxd,* positive control; *ras64B,* negative control; *E*, eye enhancer of the *white* gene. “*P*” indicates that ChIP experiments were performed with a parental transgenic line indicated above the diagram; “PΔUAS,”deletion of the UAS promoter; “PΔTs,”deletion of the 702-bp SV40 terminator; “PΔEF1,”deletion of the EF1 promoter. *Vertical lines* indicate standard deviations. All ChIP experiments were performed with heads of homozygous transgenic lines in the *w*^*1118*^ background lacking the endogenous eye enhancer and *white* gene. Background immunoprecipitation (the average normalized level after chromatin treatment with a nonspecific antibody) was subtracted from normalized specific ChIP signals (obtained with specific antibodies) at each position. Click here for file

Additional file 3: Table S1 Primers used for RT-qPCR analysis of transcripts from transgenic flies. Click here for file

Additional file 4: Table S2 Primers used for PCR in X-ChIP experiments with DNA fragments from the genome or transgenic constructs. Click here for file

Additional file 5: Figure S3 (A) Testing of Zeste antibodies by Western blot. Protein extract was prepared from wild-type (WT) or *z*^*v77h*^ larvae. *Upper panel*, antibodies against Zeste; *lower panel*, control anti-tubulin antibodies. (B) Western blot analysis of nuclear extracts (Input line), PH immunoprecipitates (PH line) and control IgG immunoprecipitates (IgG line) from Sg4 cells with antibodies against PH. (C) Western blot analysis of nuclear extracts (Input line), Sfmbt immunoprecipitates (Sfmbt line) and control IgG immunoprecipitates (IgG line) from Sg4 cells with antibodies against Sfmbt. Click here for file

Additional file 6: Supplementary methods Western blotting, preparation of the nuclear extracts and immunoprecipitation. Click here for file
