# Supplementary material for: Transcription through enhancers suppresses their activity in Drosophila
Source: Epigenetics Chromatin. 2013 Sep 26;6:31. doi: 10.1186/1756-8935-6-31 (PMC3852481; doi:10.1186/1756-8935-6-31)
Supplement: Additional file 1: Figure S1 — Summarized results of wing and body phenotype analysis in (a) (UAS)Ey(e)YW and (b) (UASR)EyeYW transgenic lines. [file 1756-8935-6-31-S1.pdf]

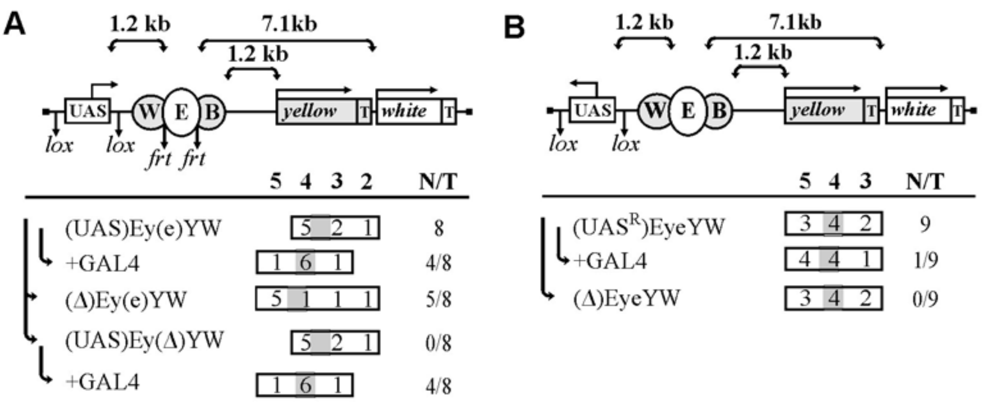

Supplementary Figure S1 Summarized results of wing and body phenotype analysis in (a) (UAS)Ey(e)YW and (b) (UAS^R)EyeYW transgenic lines.
