# Supplementary material for: Transcription through enhancers suppresses their activity in Drosophila
Source: Epigenetics Chromatin. 2013 Sep 26;6:31. doi: 10.1186/1756-8935-6-31 (PMC3852481; doi:10.1186/1756-8935-6-31)
Supplement: Additional file 2: Figure S2 — Results of ChIP with antibodies to (a) Ph and (b) Sfmbt. Diagrams summarize the results of ChIP with specific antibodies followed by real-time PCR. The ordinate shows the percentage of target sequences in the immunoprecipitated material relative to the input (10% of total cross-linked chromatin), with the genome regions for which DNA enrichment was tested being indicated on the abscissa: bxd, positive control; ras64B, negative control; E, eye enhancer of the white gene. “P” indicates that ChIP experiments were performed with a parental transgenic line indicated above the diagram; “PΔUAS,”deletion of the UAS promoter; “PΔTs,”deletion of the 702-bp SV40 terminator; “PΔEF1,”deletion of the EF1 promoter. Vertical lines indicate standard deviations. All ChIP experiments were performed with heads of homozygous transgenic lines in the w1118 background lacking the endogenous eye enhancer and white gene. Background immunoprecipitation (the average normalized level after chromatin treatment with a nonspecific antibody) was subtracted from normalized specific ChIP signals (obtained with specific antibodies) at each position. [file 1756-8935-6-31-S2.pdf]

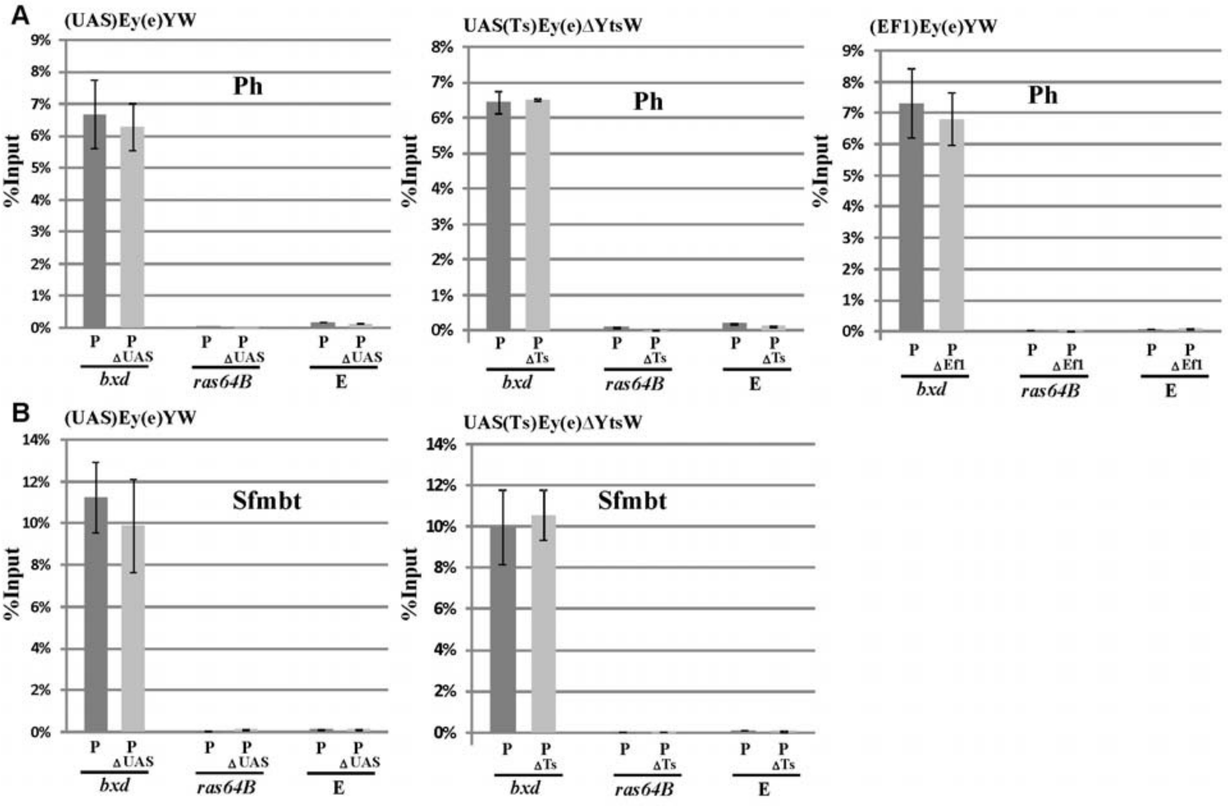

Supplementary Figure S2 Results of ChIP with antibodies to (a) Ph and (b) Sfmbt. Diagrams summarize the results of ChIP with specific antibodies followed by real-time PCR. The ordinate shows the percentage of target sequences in the immunoprecipitated material relative to the input (10% of total crosslinked chromatin), with the genome regions for which DNA enrichment was tested being indicated on the abscissa: *bxd,* positive control; *ras64B,* negative control; E, eye enhancer of the *white* gene. “P” indicates that ChIP experiments were performed with a parental transgenic line indicated above diagram; “PΔUAS” – deletion of the UAS promoter; “PΔTs” – deletion of the 702-bp SV40 terminator; “PΔEF1” – deletion of the EF1 promoter. Vertical lines indicate standard deviations. All ChIP experiments were performed with heads of homozygous transgenic lines in the w^{1118} background lacking the endogenous eye enhancer and *white* gene. Background immunoprecipitation (the average normalized level after chromatin treatment with a nonspecific antibody) was subtracted from normalized specific ChIP signals (obtained with specific antibodies) at each position.
