# Supplementary material for: Transcription through enhancers suppresses their activity in Drosophila
Source: Epigenetics Chromatin. 2013 Sep 26;6:31. doi: 10.1186/1756-8935-6-31 (PMC3852481; doi:10.1186/1756-8935-6-31)
Supplement: Additional file 3: Table S1 — Primers used for RT-qPCR analysis of transcripts from transgenic flies. [file 1756-8935-6-31-S3.pdf]

Supplementary Table S1 Primers used for RT-qPCR analysis of transcripts from transgenic flies

**Primer name**	**Sequence**
RT-1-2-forward	5'-AAATGATTATCGCCCGATTACCACA-3'
RT-1-2-reverse	5'-TCAGTGCTCGAAAAAGCTTAGTGGA-3'
RT-3-4-forward	5'-CCGATTCGAGCTCCACTCAACC-3'
RT- 3-4-reverse	5'-GGAGCGCGCTAAGTGGTCAAAA-3'
RT-5-6-forward	5'-ATCCAGTTGATTTTCAGGGACCA-3'
RT-5-6-reverse	5'-TTGGCAGGTGATTTTGAGCATAC-3'
RT-7-8-forward	5'-GCACTGGATATCATTGAACTTATCTG-3'
RT-7-8-reverse	5'-TGGACAGAGAAGGAGGCAAACA-3'
white RT-forward	5'-GCAAATGTCAGCACACGATCAT-3'
white RT-reverse	5'-GTGGGCTCATCGCAGATCA-3'
RT-9-10-forward	5'-ACTGCATTCTAGTTGTGGTTTGTCC-3'
RT-9-10-reverse	5'-CAGCGCTGACTTTGAGTGGAATG-3'
RT-11-12-forward	5'-AAGCTTATCGGGGCTGCAGGAAT-3'
RT-11-12-reverse	5'-TCAGTGCTCGAAAAAGCTTAGTGGA-3'
RT-13-14-forward	5'-TAGATCGTCAAATAAAGTCCCTA-3'
RT-13-14-reverse	5'-GTTTGGTATGATTTTTGGCCTTC-3'
yellow RT-forward	5'-TGTGACCCTGATCACCTTGG-3'
yellowRT -reverse	5'-TCGGGTATTCGGGAAAGCA-3'
Ras64B-forward	5'-GAGGGATTCCTGCTCGTCTTCG-3'
Ras64B-reverse	5'-GTCGCACTTGTTACCCACCATC-3'
