# Supplementary material for: Transcription through enhancers suppresses their activity in Drosophila
Source: Epigenetics Chromatin. 2013 Sep 26;6:31. doi: 10.1186/1756-8935-6-31 (PMC3852481; doi:10.1186/1756-8935-6-31)
Supplement: Additional file 4: Table S2 — Primers used for PCR in X-ChIP experiments with DNA fragments from the genome or transgenic constructs. [file 1756-8935-6-31-S4.pdf]

Supplementary Table S2 Primers used for PCR in X-ChIP experiments with DNA fragments from the genome or transgenic constructs

**Primer name**	**Sequence**
pUbx- forward	5'-TCCTTCCTCGCGAATGAATGAACG-3'
pUbx-reverse	5'-TCGAACGAATGTGGCACCAAATC-3'
Ras64B-forward	5'-GAGGGATTCCTGCTCGTCTTCG-3'
Ras64B-reverse	5'-GTCGCACTTGTTACCCACCATC-3'
E- forward	5'-GCGACAGGCGAGTGACAATAA-3'
E-reverse	5'-TTGGTTGAGTGGAGCTCGAATC-3'
pW- forward	5'-GCACTGGATATCATTGAACTTATCTG-3'
pW- reverse	5'-TGGACAGAGAAGGAGGCAAACA-3'
codW- forward	5′-GCAAATGTCAGCACACGATCAT-3'
codW- reverse	5′-GTGGGCTCATCGCAGATCA-3'
bxd- forward	5'-AAGAGCAAGGCGAAAGAGAGC-3'
bxd- reverse	5'-CGTTTTAAGTGCGACTGAGATGG-3'
