# Supplementary material for: Transcription through enhancers suppresses their activity in Drosophila
Source: Epigenetics Chromatin. 2013 Sep 26;6:31. doi: 10.1186/1756-8935-6-31 (PMC3852481; doi:10.1186/1756-8935-6-31)
Supplement: Additional file 5: Figure S3 — (A) Testing of Zeste antibodies by Western blot. Protein extract was prepared from wild-type (WT) or zv77h larvae. Upper panel, antibodies against Zeste; lower panel, control anti-tubulin antibodies. (B) Western blot analysis of nuclear extracts (Input line), PH immunoprecipitates (PH line) and control IgG immunoprecipitates (IgG line) from Sg4 cells with antibodies against PH. (C) Western blot analysis of nuclear extracts (Input line), Sfmbt immunoprecipitates (Sfmbt line) and control IgG immunoprecipitates (IgG line) from Sg4 cells with antibodies against Sfmbt. [file 1756-8935-6-31-S5.pdf]

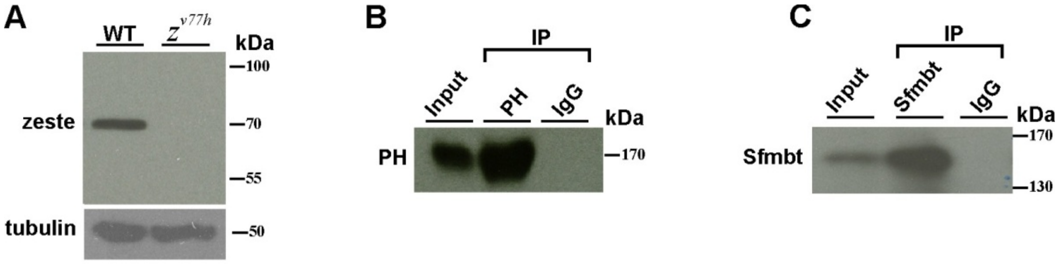

Supplementary Figure S3. (**A**) Testing of Zeste antibodies by Western blot. Protein extract was prepared from wild-type (WT) or z^{v77h} larvae. Upper panel – antibodies against Zeste; lower panel – control anti-tubulin antibodies. (**B**) Western blot analysis of nuclear extracts (Input line), PH immunoprecipitates (PH line) and control IgG immunoprecipitates (IgG line) from Sg4 cells with antibodies against PH. (**C**) Western blot analysis of nuclear extracts (Input line), Sfmbt immunoprecipitates (Sfmbt line) and control IgG immunoprecipitates (IgG line) from Sg4 cells with antibodies against Sfmbt.
