# Supplementary material for: Transcription through enhancers suppresses their activity in Drosophila
Source: Epigenetics Chromatin. 2013 Sep 26;6:31. doi: 10.1186/1756-8935-6-31 (PMC3852481; doi:10.1186/1756-8935-6-31)
Supplement: Additional file 6: Supplementary methods — Western blotting, preparation of the nuclear extracts and immunoprecipitation. [file 1756-8935-6-31-S6.pdf]

## Supplementary methods

### Western blotting

Protein extracts were prepared from 20 wild-type or $z^{v77h}$ mutant larvae ($z^{v77h}w^{67c23}$, Bloomington Center #1385). Larvae were homogenized in HEMGN buffer (25mM Hepes-KOH pH 7.6, 0.1 mM EDTA, 12.5 mM MgCl2, 10% glycerol, 0.1% NP40, 1 mM DTT, 0.1 mM PMSF, 0.3 M KCl). An equal volume of 2X SDS reducing buffer was added and then boiled for 5 minutes. The extract was centrifuged at 12 000 g for 2 min, supernatant was used for SDS-PAGE. After electrophoresis proteins were transferred onto a polyvinylidene difluoride (PVDF) membrane. The membranes were incubated for 1 h with the indicated primary antibody, followed by incubation for 1 h with the secondary antibody (GE Healthcare Amersham), which was conjugated to horseradish peroxidase. Proteins were detected using chemiluminescence reagents. As a control anti-tubulin mouse antibody (Abcam ab44928) was used.

### Preparation of the nuclear extracts and immunoprecipitation

The nuclear extracts were obtained from Drosophila Sg4 cells. For this purpose, $10^8$ cells were washed twice in 10 mL of ice cold PBS and resuspended in in 10 mL of ice cold IP-Sucrose buffer (10 mM Tris, pH 7.5; 10 mM NaCl, 10 mM MgCl2, 1mM EDTA, 1 mM EGTA, 1 mM DTT, 250 mM sucrose, EDTA-free protease inhibitor cocktail), incubated on ice for 10 min, and homogenized with a Dounce loose pestle. The nuclei were then pelleted by centrifugation at 3000 g, 40C for 10 min. The pellet was resuspended in 1 mL RIPA buffer (50 mM Tris-HCl pH 8.0, 150 mM NaCl, 2mM EDTA, 1% NP-40, 0.5% sodium deoxycholate, 0.1% SDS, EDTA-free protease inhibitor cocktail). Lysates were cleared by centrifugation at 18 000 g, + 4$^{o}$C for 10 min. Immunoprecipitation experiments involved incubation with corresponding antibody (rabbit antibody against PH, Sfmbt or nonimmune rabbit IgG) and A-Sepharose beads (Thermo Scientific). Antibody–protein complexes were collected by centrifugation. The beads were

washed three times with RIPA buffer. After the last washing step, the beads were resuspended in SDS–PAGE loading buffer, boiled, and analyzed by Western blotting.
